# Surgical Management of Palatal Placed, Inverted, Dilacerated and Impacted Mesiodens

**DOI:** 10.5005/jp-journals-10005-1038

**Published:** 2009-04-26

**Authors:** NB Nagaveni, ND Shashikiran, VV Subba Reddy

**Affiliations:** 1 Assistant Professor, Department of Pediatric Dentistry, College of Dental Sciences, Davangere-577004, Karnataka, India; 2 Professor, Department of Pediatric Dentistry, College of Dental Sciences, Davangere-577004, Karnataka, India; 3 Professor and Head, Department of Pediatric Dentistry, College of Dental Sciences, Davangere-577004, Karnataka, India

**Keywords:** Supernumerary, mesiodens, dilaceration, palatal impaction.

## Abstract

Teeth may vary in size, shape and number. Mesiodens is the most commonly occurring supernumerary tooth, usually seen between upper two central incisors which may be impacted or erupted. The present paper describes a rare case of palatal placed, inverted, severely dilacerated and impacted mesiodens which was detected on radiographic examination for some other problem.

## INTRODUCTION

In pediatric dentistry, we come across numerous anomalies in the size, shape, number and eruption of teeth. Some anomalies which are erupted in the oral cavity may be detected through routine checkup. But on the other hand, some may remain impacted within the bone, without causing any signs and symptoms. The detection of such anomalies will come into picture only while diagnosing some other problems. Here is such a case describing the presence of inverted, palatal placed, impacted asymptomatic mesiodens without patient awareness, which was detected on radiographic examination for some other problem.

The most commonly occurring supernumerary tooth is the mesiodens. This term is used to refer to an unerupted supernumerary tooth in the central region of the premaxilla between the two central incisors.^1^ The cause may be due to complex interaction of genetic and environmental factors.^2^ In Caucasian population the incidence of mesiodens is 0.3 to 0.8% for deciduous teeth and 0.15 to 3% for permanent teeth. It is most frequently found in males than females in the proportion of 2:1.^1^,^3^

Mesiodens may be impacted or erupted. It may remain in position for many years, without clinical manifestations. Sometimes complications can be seen associated with them such as impaction, delayed eruption, ectopic eruption, crowding, diastema, and eruption into the nasal floor, formation of primordial or follicular cyst with bone destruction, pain and swelling at the site and resorption of the adjacent root. Thus, early detection and removal of mesiodens is very important to prevent such complications.

## CASE REPORT

**Fig. 1.**
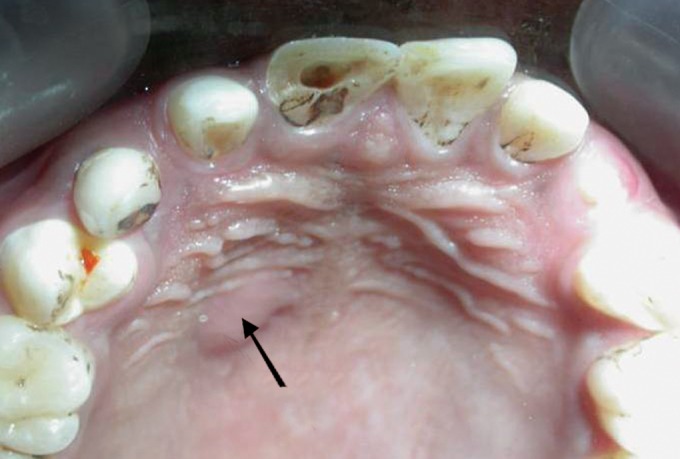
Intraoral photograph showing Ellis class IV fracture of 21 and palatal swelling with pus discharge (black arrow)

**Fig. 2.**
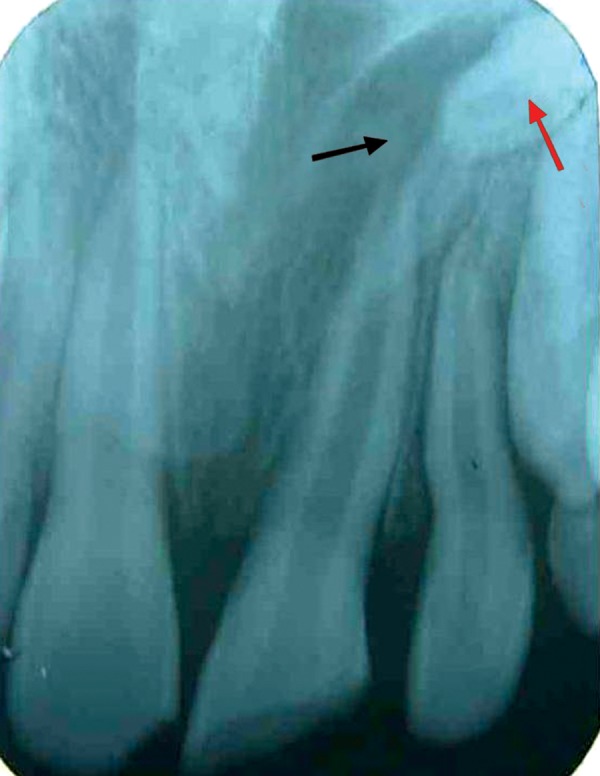
Periapical radiograph showing cystic lesion in relation to 21 (black arrow) and ‘mesiodens’ like supernumerary tooth placed in the mid-palatal region, close to the root apex of 21(red arrow)

**Fig. 3.**
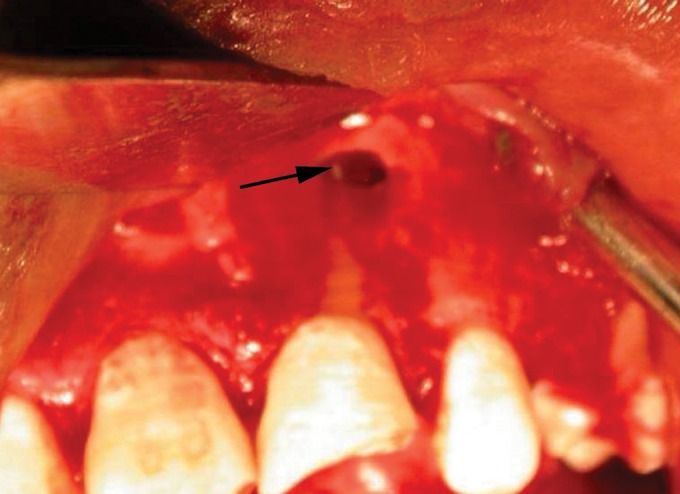
Photograph showing surgical exposure of the cystic lesion of 21. Root apex and extruded gutta percha can be seen in cystic cavity (black arrow)

**Fig. 4.**
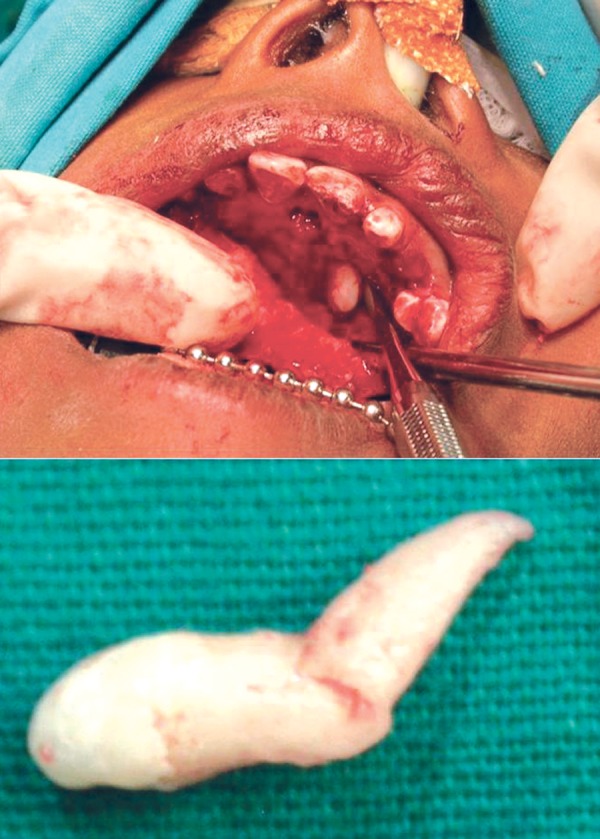
Surgical exposure for removal of mesiodens (above). Extracted mesiodens with short crown and severely dilacerated root (below)

A 12-year-old male patient reported to the Department of Pedodontics, College of Dental Sciences, Davangere, complaining of pain in the upper front tooth since 10 days. There was a history of trauma to the same tooth about 2 years back. On clinical examination, Ellis class IV fracture was found with 21 and a palatal swelling with pus discharge (Fig. 1). Periapical radiographic examination revealed a large periapical radiolucency lined by a thin radiopaque line with respect to 21 (Fig. 2). Surprisingly there was a small tooth like structure found close to the root apex of the 21, which was inverted, root of that tooth found to be dilacerated (Fig. 2). To know the proper position of this tooth a SLOB (same lingual opposite buccal) rule was used, which revealed that, the tooth was placed palatally. A diagnosis of radicular cyst and mesiodens was made. Treatment plan of intentional over obturation followed by apicectomy and surgical extraction of mesiodens under general anesthesia was made. For apicectomy of 21 labial approach was used (Fig. 3) and as the mesiodens was placed palatal, palatal entry was used for its removal (Fig. 4).

## DISCUSSION

Morphologically, the mesiodens appear as a rudimentary tooth with a cone-shaped crown, smooth surface and smaller size than the normal teeth. Sometimes, it may present with a tuberculate shape and normal size, or may be found to mimic a natural tooth. The root is generally fully formed and is often found globular. The mesiodens is most frequently found between the upper central incisors, in particular on the palatine side, along the sagittal median plane, which gives it its name.^4^

The present case is unique in several aspects. First the occurrence of mesiodens is very rare in the mid palatal region. The crown of mesiodens was very small in size with conical shape. The root was fully developed having a severe dilaceration (almost with 90° bend). Usually all mesiodens have conical crown with short conical root. There are no reports citing the dilacerated mesiodens. It appears that the present case is the first report of mesiodens with dilacerated root. In addition, the placement of the tooth was again very unusual with the crown facing posterior and the root anterior. Finally, it is either associated with neither any clinical symptoms, nor any developmental abnormalities or syndromic features in a patient. It seems that due to erupting force of the central incisor the tooth might have shifted to mid-palatal region and also due to continued pressure from the erupting central incisor the root might have dilacerated.

Since in most cases, the mesiodens is totally impacted (88.7%),^5^,^6^ it may cause a delay or even prevent the central incisors from erupting, whereas, when it erupts normally, it shifts towards the area that should be occupied by the permanent tooth and can determine the dislocation of the adjacent elements that will be subject to diastema and/or malpositioned. At times, by erupting in the palate it causes the early loss of one or more deciduous teeth as a result of root resorption. The mesiodens which is seen in the nasal cavity may evolve into cystic forms or they may erupt.^3^ Considering all these complications that arise from the mesiodens, extraction of the same was done.

The increased frequency of mesiodens among developmental anomalies, its deleterious effects on normal functioning, sometimes their asymptomatic nature when they are impacted, emphasizes the importance of radiographic examination of all children. Early diagnosis assists early intervention, more favorable prognosis and minimal complications.

The cystic lesion was found in relation to both 21 and mesiodens. It was a diagnostic dilemma whether it is a radicular cyst due to periapcial infection of 21 or a dentigerous cyst arising from mesiodens. As majority of mesiodens lead to a cyst formation, the possibility of development of dentigerous cyst should be considered. But the patient’s history of trauma, clinical features consisting of long standing necrosis of 21, were strongly suggested that, cyst was a radicular rather than a dentigerous. Moreover, if it would be a dentigerous, the cyst should surround the entire crown of the mesiodens. But the crown of the mesiodens found outside the cystic cavity excluding the diagnosis of dentigerous cyst.

From the present case report it was concluded that, because of the increased frequency of mesiodens and also its possibility of atypical location without any clinical manifestations for many years, justify the radiographic examination of every child.

## References

[1] Gallas MM, Garcia A (2000). Retention of permanent incisor by
mesiodens: a family affair.. Br Dent J.

[2] Primosch RE (1981). Anterior supernumerary teeth assessment and
surgical intervention in children.. Pediatr Dent.

[3] Kupietzky A, Rotstein  I, Kischinovsky D (2000). A multidisciplinary
approach to the treatment of an intruded maxillary permanent
incisor complicated by the presence of two mesiodens.. Pediatr
Dent.

[4] Giancotti A, Grazzini F, De Dominicis F, Romanini G, Arcuri C (2002). Multidisciplinary evaluation and clinical management of
mesiodens.. J Clin Pediatr Dent.

[5] Hurlen B, Humerfelt D (1992). Characteristic of premaxillary hyperdontia.
A radiographic study.. Aust Dent J.

[6] Grimanis GA, Kyriakides AT, Spyropoulos ND (1991). A survey on
supernumerary molars.. Quintessence Int.

